# Association of Vitamin D Levels and Systemic Inflammation in Patients with Hashimoto’s Thyroiditis

**DOI:** 10.3390/diseases14090349

**Published:** 2026-09-20

**Authors:** Dean Kaličanin, Vanna Žnidar, Ivana Listeš, Maja Cvek, Ana Barić Žižić, Marko Vuletić, Sanda Sladić, Vivian Eneas Micek, Vesela Lovrić Torlak, Ante Punda, Vesna Boraska Perica

**Affiliations:** 1 Department of Medical Biology, University of Split School of Medicine, 21000 Split, Croatia; dkalican@mefst.hr (D.K.); ivana.listes@mefst.hr (I.L.); 2 Department of Nuclear Medicine, University Hospital Split, 21000 Split, Croatia; maja.cvek.st@mail.com (M.C.); ana.baaric@gmail.com (A.B.Ž.); mavuletic@gmail.com (M.V.); sanda_gracan@yahoo.com (S.S.); veselakbsplit@yahoo.com (V.L.T.); ante.punda@gmail.com (A.P.); 3 Klinik für Unfall-, Hand- und Wiederherstellungschirurgie, Universitätsklinikum Essen, University of Duisburg-Essen, 45147 Essen, Germany; vivian-micek_1999@hotmail.de

**Keywords:** vitamin D, vitamin D deficiency, Hashimoto disease, autoimmune, inflammation, cytokines, biomarkers, proteomics, autoimmune diseases

## Abstract

**Background/Objectives**: Vitamin D is an important regulator of immune and inflammatory processes, while its deficiency is frequently observed in Hashimoto’s thyroiditis (HT). The relationship between Vitamin D and systemic inflammation in HT remains insufficiently understood, particularly across different disease stages. **Methods**: We used the Olink Target 96 Inflammation panel to investigate this association. Inflammatory proteins and serum 25(OH)D levels were measured in 257 HT patients and 173 controls from the CROHT biobank. Participants were categorized as Vitamin D deficient (<20 ng/mL) or non-deficient (≥20 ng/mL), with HT patients further stratified as euthyroid, hypothyroid, or levothyroxine-treated. Associations between 25(OH)D and 92 inflammatory proteins were assessed using multivariable linear regression adjusted for age, sex, BMI, smoking, and season of blood sampling, followed by interaction testing and meta-analysis. **Results**: Vitamin D deficiency was prevalent in both HT patients and controls. Among Vitamin D-deficient HT patients, higher 25(OH)D levels showed nominal inverse associations with the previously reported HT-associated inflammatory proteins IL-17C, CCL20, and CCL11, as well as with GDNF. Among non-deficient HT patients, a nominal positive association with CD6 was observed. None of these associations or interaction terms remained statistically significant after false discovery rate correction. **Conclusions**: These findings should therefore be considered exploratory and hypothesis-generating. Nevertheless, the observed Vitamin D status-specific patterns suggest that the relationship with inflammatory profiles in HT may differ according to Vitamin D status and warrant validation in prospective and mechanistic studies.

## 1. Introduction

Vitamin D (calciferol) is a fat-soluble prohormone essential for calcium homeostasis and bone metabolism [1]. The main forms are Vitamin D_3_, produced in the skin by UVB exposure, and Vitamin D_2_, derived from plants and fungi [2]. Both are inactive precursors that are converted in the liver to 25-hydroxyvitamin D (25(OH)D) and then in the kidney to the active form, 1,25-dihydroxyvitamin D (calcitriol) [2]. Calcitriol binds to Vitamin D receptors (VDR) to regulate gene expression, affecting not only bone health but also immune and inflammatory processes [3,4].

Current scientific guidelines recommend measuring 25(OH)D in blood as the standard test, as it reflects both, the Vitamin D produced in the skin and obtained from food and supplements, with a relatively long half-life [1]. Optimal 25(OH)D levels are still debated due to differing clinical and public health recommendations worldwide [2]. Although several organizations define Vitamin D status differently, Vitamin D deficiency is generally considered as serum 25(OH)D concentrations below 20 ng/mL. Evidence suggests that the greatest physiological and immunological effects of Vitamin D supplementation are observed in individuals who are Vitamin D deficient [5]. Adequate Vitamin D levels are essential for overall human health; however, Vitamin D deficiency has become a global problem and has been associated with various health problems [6]. For example, Vitamin D deficiency has been associated with many medical conditions such as cardiovascular, neurological, endocrine and autoimmune diseases, including Hashimoto’s thyroiditis (HT) [7,8].

HT is the most common autoimmune disorder globally, with 4–10-fold higher female predominance [9]. It is characterized by chronic inflammation of the thyroid gland, including lymphocytic infiltration and gradual destruction of thyroid tissue [10]. The disease is initiated by a loss of immune tolerance to thyroid-specific self-antigens, primarily thyroglobulin (Tg) and thyroid peroxidase (TPO) [11]. HT typically progresses from a euthyroid phase with normal thyroid function to subclinical hypothyroidism with elevated TSH but normal thyroid hormone levels, and eventually to overt hypothyroidism. The disease occurs in genetically predisposed individuals and is typically triggered by environmental factors [12]. Among environmental factors, Vitamin D has been extensively analyzed; although its causal role in HT pathogenesis remains debated [8,13], Vitamin D deficiency is frequently observed in patients with HT.

In our previous study, we observed a high prevalence of Vitamin D deficiency in HT patients, especially in HT patients with overt hypothyroidism, which is an advanced stage characterized by elevated TSH, reduced thyroid hormone levels and a more pronounced autoimmune process [14]. These findings are consistent with a large study including 25,018 controls and 27,800 individuals with HT, which also reported a higher prevalence of Vitamin D deficiency in the hypothyroid group [15].

Some studies have examined the relationship between Vitamin D and inflammation, consistently demonstrating that Vitamin D exerts anti-inflammatory effects, particularly in individuals with elevated inflammatory activity and low baseline Vitamin D levels. These effects are thought to be mediated through the modulation of immune cell activity and the regulation of cytokine production, whereby Vitamin D suppresses pro-inflammatory mediators while promoting anti-inflammatory pathways [16,17]. Such immunomodulatory properties have made Vitamin D of particular interest in the context of autoimmune diseases. In line with this, research focusing specifically on HT has reported that reduced Vitamin D levels are inversely correlated with inflammatory proteins and markers of immune activation [18,19]. These findings suggest that Vitamin D deficiency may contribute to heightened inflammatory responses in individuals with HT. However, these studies have been limited by small sample sizes and a narrow range of inflammatory markers, highlighting the need for larger and more comprehensive investigations.

Given the proposed immunomodulatory role of Vitamin D, we investigated its association with inflammatory status in HT patients and controls from the CROHT biobank, and across three disease-severity subgroups of patients [14]. In our previous study, IL-17C, CCL-20, and CCL-11 were identified as inflammatory proteins associated with HT [20]. The present study extends these findings by examining whether the levels of these and other inflammatory proteins are associated with serum Vitamin D concentrations in HT, including according to Vitamin D status and disease stage. Inflammatory status was assessed using the Olink Target 96 Inflammation panel, which enables simultaneous quantification of 92 inflammation-related proteins involved in many immune pathways, including cytokine signaling, immune response, and cellular stress [21]. This high-throughput proteomic approach provides a comprehensive assessment of systemic inflammation and allows exploration of potential immunological mechanisms linking Vitamin D with HT.

## 2. Materials and Methods

### 2.1. Subjects

For this research, serum samples and clinically relevant phenotypes from 257 adult patients with HT and 173 control participants were obtained from the CROHT biobank. Patient recruitment was carried out at the Clinical Department of Nuclear Medicine at the University Hospital of Split. HT was diagnosed according to the ETA recommendations and guidelines for the management of subclinical hypothyroidism [22]. Inclusion criteria for HT patients included: (1) an echographic pattern consistent with diffuse thyroid disease; and (2) increased thyroid-stimulating hormone (TSH) and/or decreased thyroid hormones: triiodothyronine (T3), thyroxine (T4), or free thyroxine (fT4) and/or elevated thyroid autoantibodies (TPOAb, TgAb). Control participants were confirmed to be free of HT based on clinical examination and the following criteria: (1) a homogeneous, normoechogenic thyroid parenchyma on ultrasound without diffuse or focal lesions; and (2) all thyroid function parameters within reference ranges: TSH (0.3–3.6 mIU/L), T3 (1.3–3.6 nmol/L), T4 (57.3–161 nmol/L), fT4 (10.3–22.8 pmol/L), TPOAb (1–16 IU/mL), and TgAb (5–100 IU/mL).

Vitamin D levels (25(OH)D) were measured using the LIAISON 25(OH) Vitamin D Total chemiluminescence immunoassay (DiaSorin, Saluggia, Italy). Thyroid hormone levels (TSH, FT4, FT3) and thyroid-specific antibodies (TgAb and TPOAb) were assessed using LIAISON chemiluminescence immunoassays (DiaSorin, Saluggia, Italy). All individuals using medications or dietary supplements that could affect Vitamin D levels were excluded from the study. Participants were stratified according to serum 25(OH)D concentrations into Vitamin D-deficient (<20 ng/mL) and non-deficient (≥20 ng/mL) groups, using the established threshold for Vitamin D deficiency [23].

### 2.2. Measurement of Inflammatory Proteins

The Olink Target 96 Inflammation panel employs Proximity Extension Assay (PEA) technology to simultaneously quantify 92 inflammation-related proteins (Olink™ Proteomics, Uppsala, Sweden). PEA technology uses pairs of antibodies, each conjugated to unique DNA oligonucleotides, which, upon binding to their target protein, enable the formation of a DNA barcode that is subsequently amplified and quantified by qPCR. This dual-antibody approach ensures high specificity and sensitivity, making the panel particularly suitable for detecting low-abundance proteins involved in inflammatory processes. The resulting data are reported in Normalized Protein Expression (NPX) units, which are relative, log2-scaled values normalized against internal and inter-plate controls, providing robust and reproducible measurements [21].

### 2.3. Statistical Analysis

Prior to statistical analysis, HT patients were stratified by disease stage into three groups: euthyroid (EUTHY; TSH 0.3–3.6 mIU/L), hypothyroid (HYPO; TSH > 3.6 mIU/L, therapy-naive), and those receiving levothyroxine (LT4 group). Both, the HT subgroups and the control group were further categorized by Vitamin D status into deficient (<20 ng/mL) and non-deficient (≥20 ng/mL) subgroups. This resulted in the final eight study groups: Controls, EUTHY, HYPO, and LT4, each subdivided according to Vitamin D status.

Associations between serum 25(OH)D concentration and protein expression (NPX values) were evaluated separately within the EUTHY, LT4, and HYPO groups using multivariable linear regression models. NPX values were treated as the dependent variables, while serum 25(OH)D concentration was included as the primary independent variable. All models were adjusted for age, sex, body mass index (BMI), smoking status, and season of blood sampling. Season of blood sampling was categorized into four calendar seasons (winter, spring, summer, and autumn) and included as a covariate to account for seasonal variation in sunlight/UVB exposure and Vitamin D status. For each protein, the regression coefficient (β) and its standard error (SE) for 25(OH)D were extracted from each subgroup-specific model.

These subgroup-specific effect estimates were subsequently combined using random-effects meta-analysis with restricted maximum likelihood (REML) estimation, as implemented in the rma() function of the R package metafor. Between-group heterogeneity was assessed using Cochran’s Q statistic, τ^2^, and I^2^. To facilitate biological interpretation of the meta-analytic effect estimates, regression coefficients expressed on the NPX (log2) scale were additionally transformed into relative percentage differences in protein levels associated with a 10 ng/mL higher serum 25(OH)D concentration. Relative percentage differences were calculated as (2^10β^ − 1) × 100, with the corresponding 95% confidence limits transformed using the same approach. As NPX represents relative protein expression, these estimates should be interpreted as relative differences rather than changes in absolute protein concentrations.

To formally test for effect modification, multiplicative interaction analyses were performed. Specifically, interaction terms (continuous 25(OH)D × Vitamin D status and continuous 25(OH)D × HT status) were introduced into the adjusted linear regression models to evaluate whether the associations differed significantly between subgroups.

As a sensitivity analysis, pooled linear regression models were additionally performed separately among Vitamin D-deficient and Vitamin D-sufficient HT participants, combining the EUTHY, HYPO, and LT4 groups and including disease stage (EUTHY, HYPO, or LT4) as an additional covariate alongside age, sex, BMI, smoking status, and season of blood sampling.

Statistical analyses were performed using R version 4.4.2 (R Core Team, Vienna, Austria). Because 92 inflammatory proteins were evaluated, multiple testing was addressed using the False Discovery Rate (FDR) procedure. FDR-adjusted P-values were calculated separately for the Vitamin D-deficient and Vitamin D-non-deficient HT meta-analyses. Nominal *p*-values and FDR-adjusted *p*-values are reported to distinguish exploratory nominal associations from associations that remain statistically significant after correction for multiple comparisons.

## 3. Results

The study included 257 participants with HT and 173 control participants. The Vitamin D-non-deficient group (*n* = 186) included 25 EUTHY, 45 LT4, and 50 HYPO patients, alongside 66 controls. The Vitamin D-deficient group (*n* = 244) was composed of 22 EUTHY, 48 LT4, 67 HYPO subjects, and 107 controls. The clinical characteristics for all subgroups, categorized by both disease stage and Vitamin D status, are presented in Table 1 and Table 2. There were no significant differences in thyroid-related clinical characteristics between Vitamin D-non-deficient and Vitamin D-deficient individuals within each subgroup, except for Vitamin D. Information on disease duration was available from the CROHT Biobank.

The association between proteomic biomarkers and Vitamin D levels within each disease stage and Vitamin D status subgroup is shown in Supplementary Table S1. No significant results were found within the control group, whether in the Vitamin D-non-deficient or deficient groups. We further focused on the meta-analysis across HT patients only. This approach allowed us to investigate the relationship between Vitamin D and inflammatory proteins within HT while accounting for the distinct disease-stage groups and combining their subgroup-specific effect estimates. All meta-analysis results are provided in Supplementary Table S2, while nominally significant results are presented in Table 3.

The meta-analysis across Vitamin D-deficient HT subgroups (EUTHY, LT4, and HYPO) demonstrated that higher Vitamin D levels were nominally associated with lower concentrations of several inflammatory markers (Table 3): Glial cell line-derived neurotrophic factor (GDNF), Interleukin-17C (IL-17C), C-C motif chemokine ligand 11 (CCL-11), and C-C motif chemokine ligand 20 (CCL-20). To facilitate interpretation of the magnitude of these associations, the meta-analytic effect estimates were additionally expressed as relative percentage differences in protein levels associated with a 10 ng/mL higher serum 25(OH)D concentration (Supplementary Table S3). For example, a 10 ng/mL higher 25(OH)D concentration was associated with an estimated 29.7% lower relative IL-17C level (95% CI, 48.0% lower to 5.0% lower) among vitamin D-deficient patients with HT.

In contrast to the deficient cohort, the meta-analysis of HT patients with non-deficient Vitamin D levels revealed a nominal positive association between Vitamin D concentration and CD6 levels (*p* = 0.038). None of these associations remained statistically significant after FDR correction.

To evaluate whether the observed differences between the stratified subgroups represented true effect modification, formal interaction analyses were conducted. While nominal interactions were detected for several proteins across the functional subgroups (four in EUTHY, three in HYPO, and two in LT4) and between HT patients and controls (IL-4, CCL-23, and FGF-23), none of these interaction terms remained statistically significant after FDR correction. Consequently, these stratified findings should be interpreted with caution as exploratory and hypothesis-generating.

As a sensitivity analysis, pooled regression models combining the EUTHY, HYPO, and LT4 groups were performed separately for Vitamin D-deficient and Vitamin D-sufficient participants, with disease stage included as an additional covariate. Among Vitamin D-deficient participants, the four proteins identified in the subgroup-based meta-analysis (GDNF, IL-17C, CCL-11, and CCL-20) showed nominal associations with Vitamin D in the pooled analysis, with consistent directions and similar effect estimates (β = −0.020, −0.056, −0.028, and −0.055, respectively). Among Vitamin D-sufficient participants, the association with CD6 observed in the subgroup-based meta-analysis was not reproduced in the pooled analysis. Additional nominal associations with IL-6 in Vitamin D-deficient participants and IFN-γ and OPG in Vitamin D-sufficient participants were observed. None of these associations remained statistically significant after FDR correction. Full results of the pooled sensitivity analyses are provided in Supplementary Table S4.

## 4. Discussion

Given that the immunomodulatory role of Vitamin D in HT is not yet fully elucidated, this study aimed to clarify how Vitamin D status relates to the complex landscape of systemic inflammatory markers in patients with HT. An exploratory finding of this study was the observation of distinct subgroup-specific patterns according to Vitamin D status. Specifically, nominal inverse associations with previously reported HT-associated inflammatory proteins, including IL-17C, CCL20, and CCL11 [20], were observed among vitamin D-deficient patients. However, formal interaction testing did not provide statistically significant evidence of differences between Vitamin D-deficient and non-deficient groups after correction for multiple testing. Therefore, these findings should not be interpreted as evidence of a biological threshold at 20 ng/mL, but rather as exploratory subgroup-specific patterns that warrant further investigation. Previous studies have suggested that the effects of Vitamin D may vary according to baseline Vitamin D status, with greater clinical benefits reported in individuals with lower baseline levels [5]. Nevertheless, the present data cannot determine whether the observed subgroup-specific patterns reflect true biological differences or other factors, including statistical variability. These findings should therefore be considered hypothesis-generating and require confirmation in adequately powered prospective and mechanistic studies.

In contrast, when Vitamin D deficiency was not present, the observed associations were less pronounced, possibly indicating that further increases in Vitamin D levels are less strongly related to these inflammatory markers. This observation is consistent with reported non-linear dose–response curves for Vitamin D’s extra-skeletal effects, although such patterns cannot be established from the present cross-sectional data [24]. Furthermore, the absence of significant associations between vitamin D and inflammatory proteins in the control group may suggest that the observed patterns are related to the underlying autoimmune context of HT. These results are consistent with previous evidence indicating that Vitamin D–related immunomodulation is more pronounced in inflammatory or autoimmune settings compared to the homeostatic state of healthy individuals [6,18].

### 4.1. Vitamin D Deficiency and Inflammatory Markers in HT

A meta-analysis encompassing Vitamin D–deficient HT patients showed nominal inverse associations between Vitamin D levels and concentrations of four proteins: GDNF, IL-17C, CCL-20 and CCL-11.

The strongest association was found for GDNF protein (Table 3). GDNF is a well-characterized neurotrophic factor promoting neuronal survival and repair, and recently its family ligands have been shown to participate in peripheral inflammatory responses beyond the nervous system, with several immune and epithelial cells capable of expressing GDNF family ligands and receptors during inflammation and tissue injury [25]. GDNF expression has also been observed in inflammatory infiltrates in autoimmune neural tissue, supporting its involvement in localized inflammatory processes [26]. Although GDNF has not been directly linked to HT, it has been associated with papillary thyroid carcinoma [27].

The observed inverse association between GDNF and Vitamin D, specifically within the Vitamin D-deficient cohort, may reflect a compensatory response to heightened systemic inflammation. While GDNF is a well-established neurotrophic factor involved in neuronal survival and synaptic plasticity, its increased circulating levels in Vitamin D-deficient patients may represent a response to inflammatory processes rather than a direct effect of Vitamin D deficiency. However, because the present study did not assess the tissue source of circulating GDNF or its functional role in HT, these interpretations remain speculative and should be considered hypothesis-generating, requiring confirmation in mechanistic and longitudinal studies. In contrast to GDNF, which remains less explored in thyroid pathology, the remaining three proteins identified in our analysis, IL-17C, CCL-20, and CCL-11, are more directly related to established inflammatory mechanisms of HT.

The finding of negative associations of IL-17C, CCL-20, and CCL-11 proteins with Vitamin D levels in Vitamin D-deficient patients with HT is particularly noteworthy in the context of our previous comprehensive study of inflammatory proteins and HT [20]. While our previous study identified IL-17C, CCL-20, and CCL-11 as inflammatory proteins associated with HT, the present study extends these findings by demonstrating nominal associations between these HT-related proteins and Vitamin D levels, particularly among Vitamin D-deficient patients. Thus, the present findings provide a potential link between Vitamin D status and inflammatory protein profiles previously associated with HT.

In more detail, we previously identified IL-17C as the most significantly associated protein with HT, which exhibits a steady and significant increase from HT’s onset through its advanced stages [20]. IL-17C is released in response to pro-inflammatory cytokines and bacterial infections to initiate rapid innate immune responses in epithelial tissues. It operates via an autocrine signaling mechanism through the IL-17RA/IL-17RE receptor complex, triggering the production of inflammatory mediators, chemokines, and antimicrobial peptides. Furthermore, it acts as a direct regulator of occludin expression in epithelial cells, highlighting its critical role in maintaining mucosal barrier integrity [28]. Our results of a negative association of Vitamin D with IL-17C are in line with previous findings in other autoimmune diseases. In multiple sclerosis, Vitamin D was found to significantly reduce pro-inflammatory Th17-related cytokines [29], whereas Vitamin D3 was found to significantly decrease IL-17A levels in patients with asthma [30]. Similarly, the combination of Vitamin D3 and all-trans retinoic acid has a synergistic effect in suppressing both the development and proliferation of Th17 cells [31].

The second pro-inflammatory protein, CCL-20, was also found in our previous study to be significantly increased in hypothyroid HT patients [20]. The finding of CCL-20 is noteworthy as it acts as a chemoattractant for Th17 cells because it interacts with the CCR6 receptor expressed on Th17 cells, including those producing IL-17C, facilitating a critical shift where regulatory T cells (Tregs) differentiate into a pathogenic Th17 lineage [32,33]. This mechanism has been proposed to contribute to a shift from immune tolerance toward chronic inflammation. In line with our observations of a negative correlation between Vitamin D and CCL-20, similar results have been reported in patients with ulcerative colitis [34]. In addition, administration of Vitamin D in experimental autoimmune encephalomyelitis resulted in downregulation of both IL-17 and CCL-20 [35]. Vitamin D3 was also found to decrease CCL-20 in corneal cells [36].

Finally, CCL-11 was the third identified protein in the current study that was found to be a HT-associated protein and a marker for distinguishing early from hypothyroid HT in our previous study [20]. CCL-11 is primarily known for eosinophilic recruitment in allergic and certain autoimmune conditions. Our findings align with results of a randomized controlled clinical trial where patients with type 2 diabetes showed downregulation of CCL-11 upon treatment with Vitamin D supplementation [37], similarly as did the patients with chronic lymphocytic leukemia [38], asthma [39] and chronic rhinosinusitis with nasal polyps [40].

In conclusion, the results from our current study suggest that higher Vitamin D levels are associated with lower concentrations of several HT-related inflammatory proteins in Vitamin D-deficient patients (IL-17C, CCL-20, and CCL-11). These findings support further investigation of Vitamin D deficiency as a potential modifier of immune profiles in HT. However, because this was a cross-sectional observational study, no conclusions can be drawn regarding the effectiveness of Vitamin D supplementation, which should be evaluated in prospective interventional studies.

Studies conducted in different populations have reported varying associations between Vitamin D status and inflammatory markers in HT. In a Brazilian cohort, Vitamin D levels were positively correlated with IL-17, TNF-α, and IL-5 in patients with HT [41]. Although IL-17A, TNF, and IL-5 were also assessed in our study, none showed a significant association with Vitamin D; instead, we identified an inverse association with IL-17C specifically in Vitamin D-deficient patients. Similarly, one other study reported lower Vitamin D concentrations and higher IL-1β, IL-8, IL-10, and TNF-α levels in patients with HT [19]. In our cohort, IL-8, IL-10, and TNF were assessed but were not significantly associated with Vitamin D, while IL-1β was not included in our panel. Thus, although previous studies support an association between Vitamin D status and inflammatory activity in HT, the specific inflammatory markers involved differ between cohorts, potentially reflecting differences in population and clinical characteristics, Vitamin D status, analytical methodology, and patient stratification. Our findings extend these observations by identifying IL-17C, CCL-20, CCL-11, and GDNF as Vitamin D-associated proteins specifically in Vitamin D-deficient patients with HT.

### 4.2. Vitamin D Non-Deficiency and Inflammatory Markers in HT

Our meta-analysis revealed a nominal positive association between circulating Vitamin D levels and CD6 across Vitamin D-non-deficient HT patients. CD6 is a costimulatory surface glycoprotein expressed on mature T lymphocytes that contributes to T-cell activation, proliferation, and immune synapse stabilization through interactions with its ligand, activated leukocyte cell adhesion molecule (ALCAM) on antigen-presenting cells [42,43]. Beyond its role in antigen-specific immune responses, CD6-mediated signaling regulates T-cell activation, differentiation, and migration, thereby shaping adaptive immune responses and maintaining immune homeostasis [42,43]. Dysregulation of CD6 signaling has been implicated in several autoimmune diseases, including multiple sclerosis and rheumatoid arthritis, underscoring its role in modulating immune tolerance [42,43].

Experimental evidence further indicates that Vitamin D, through activation of the Vitamin D receptor (VDR), can modulate T-cell surface phenotype, including upregulation of CD6, while reducing the expression of other activation-associated markers [44].

The nominal positive association observed between Vitamin D and CD6 levels in Vitamin D-non-deficient patients may reflect a distinct regulatory pattern of immune-related proteins. In this specific context, Vitamin D appears linked to markers of T-cell homeostasis, where its sufficiency could support higher CD6 levels involved in immune signaling and the maintenance of self-tolerance.

### 4.3. Strengths and Limitations

A major strength of this study is that, to our knowledge, it represents the most comprehensive investigation to date examining the relationship between Vitamin D status and a broad spectrum of inflammatory markers in patients with HT. By utilizing high-throughput Olink proteomics technology, we were able to simultaneously assess a large panel of inflammatory proteins with high sensitivity and specificity. An additional strength is the well-characterized cohort of patients from the CROHT biobank, stratified according to Vitamin D deficiency status, which enabled the evaluation of associations within clinically relevant subgroups.

Despite these strengths, several limitations should be acknowledged. First, the cross-sectional design precludes inferences of causality between Vitamin D status and the observed inflammatory protein profiles. Second, although the meta-analysis highlighted consistent trends, the smaller sample size within certain subgroups may limit the generalizability of our findings. Furthermore, because these stratified results rely on nominal associations that did not survive strict FDR correction, they should be interpreted as exploratory. Nevertheless, these distinct patterns offer valuable, hypothesis-generating insights that warrant validation in future prospective, longitudinal, and mechanistic studies. Third, residual confounding cannot be excluded. Although our regression models accounted for age, sex, BMI, smoking status, and season of blood sampling, other factors that may influence circulating 25(OH)D concentrations and inflammatory profiles were not comprehensively available for adjustment. These include dietary habits and Vitamin D intake, alcohol consumption, physical activity, and sun exposure. Additionally, while our previous data [14] indicated only a weak correlation between Vitamin D and TSH, residual confounding from thyroid hormone intake levels or clinical thyroid status cannot be entirely ruled out. Such factors may affect Vitamin D status through differences in dietary intake, lifestyle, and endogenous Vitamin D synthesis and could therefore contribute to residual confounding. Additionally, because medical phenotypes were self-reported, a risk of underreported or undiagnosed conditions (e.g., diabetes or celiac disease) cannot be entirely ruled out. To mitigate this, we cross-referenced survey data with the participants’ medication records, revealing no hidden cases that could significantly skew our findings. Finally, the present study assessed circulating 25(OH)D and inflammatory proteins at a single time point, and therefore cannot determine whether changes in Vitamin D status precede or follow changes in inflammatory pathways.

## 5. Conclusions

This study identified exploratory patterns linking Vitamin D levels to specific inflammatory markers in HT across different Vitamin D strata. In Vitamin D-deficient patients, higher 25(OH)D concentrations were nominally associated with lower levels of the neurotrophic factor GDNF and inflammatory drivers, namely IL-17C, CCL-20, and CCL-11. These findings suggest that immune-related pathways may be associated with Vitamin D variations in states of deficiency. Conversely, in Vitamin D-non-deficient patients, a nominal positive correlation was observed with CD6, pointing toward a distinct regulatory pattern once sufficiency is reached. Although these subgroup-specific findings did not maintain statistical significance after stringent FDR correction and must be viewed as exploratory, they highlight Vitamin D status as a potential modulator of immune profiles in HT. Ultimately, given our cross-sectional design, no definitive conclusions can be drawn regarding the efficacy of Vitamin D supplementation. Future prospective and interventional studies are warranted to determine whether correcting Vitamin D deficiency can actively modify these inflammatory pathways or improve clinical outcomes in HT.

## Figures and Tables

**Table 1:** Baseline thyroid-related characteristics of patients with HT and control participants.

Parameter	Control (*n* = 173)	HT (*n* = 257)	*p* Value
Age (years)	35.96 (30.43–45.27)	37.73 (29.42–47.66)	0.967
Female, *n* (%)	163 (94.2)	238 (92.6)	0.513 *
BMI (kg/m^2^)	22.49 (20.90–25.35)	23.39 (20.83–26.89)	0.0793
BSA (m^2^)	1.78 (1.69–1.92)	1.80 (1.69–1.94)	0.2141
Thyroid volume, cm^3^	8.62 (6.66–10.59)	10.59 (7.47–14.56)	<0.001
T3, nmol/L	1.50 (1.40–1.70)	1.60 (1.30–1.80)	0.2291
T4, nmol/L	101.00 (89.30–117.00)	101.00 (87.70–117.00)	0.9971
fT4, pmol/L	12.70 (11.90–13.70)	11.90 (10.10–13.10)	<0.001
TSH, mIU/L	1.49 (1.14–1.96)	4.11 (2.11–7.13)	<0.001
TgAb, IU/mL	10.60 (9.00–17.10)	212.00 (85.50–569.00)	<0.001
TPOAb, IU/mL	3.20 (1.20–8.10)	361.00 (104.20–750.00)	<0.001
Vitamin D, ng/mL	16.80 (12.80–22.70)	19.10 (14.30–24.80)	0.0241

All *p*-values were calculated using the Wilcoxon rank-sum test, whereas *p* * was calculated using Pearson’s chi-square test. Abbreviations: BMI, body mass index; BSA, body surface area; T3, triiodothyronine; T4, thyroxine; fT4, free thyroxine; TSH, thyroid-stimulating hormone; TgAb, thyroglobulin antibodies; TPOAb, thyroid peroxidase antibodies. Data are shown as median (IQR).

**Table 2:** Baseline thyroid-related characteristics of the three groups of patients with HT according to Vitamin D status.

Parameter	EUTHY Deficient	EUTHY Non Deficient	*p*	HYPO Deficient	HYPO Non Deficient	*p*	LT4 Deficient	LT4 Non Deficient	*p*
	(*n* = 22)	(*n* = 25)		(*n* = 67)	(*n* = 50)		(*n* = 48)	(*n* = 45)	
Age (years)	34.03 (22.03–46.07)	33.84 (25.51–39.88)	0.789	39.18 (31.23–50.36)	35.59 (30.05–43.24)	0.181	44.27 (31.07–50.90)	37.73 (28.63–46.45)	0.143
BMI (kg/m^2^)	23.51 (21.55–26.44)	22.10 (20.62–25.21)	0.311	23.57 (21.00–28.71)	23.38 (20.73–26.36)	0.253	23.63 (20.83–26.45)	23.46 (20.76–26.99)	0.587
BSA (m^2^)	1.73 (1.71–1.91)	1.77 (1.64–1.86)	0.436	1.80 (1.69–1.98)	1.81 (1.70–1.97)	0.866	1.78 (1.67–1.91)	1.87 (1.73–1.96)	0.092
Thyroid volume, cm^3^	12.22 (9.86–17.18)	13.22 (9.89–15.33)	0.957	9.56 (7.07–14.65)	11.05 (9.22–14.55)	0.201	8.79 (6.37–12.69)	8.67 (6.87–11.99)	0.854
T3, nmol/L	1.50 (1.33–1.70)	1.70 (1.50–1.90)	0.056	1.50 (1.30–1.80)	1.50 (1.12–1.78)	0.569	1.70 (1.50–1.80)	1.50 (1.30–1.80)	0.403
T4, nmol/L	96.10 (87.33–112.75)	94.80 (87.70–111.00)	0.822	93.10 (81.75–108.00)	94.90 (80.62–114.50)	0.600	110.00 (101.00–125.25)	115.00 (98.90–134.00)	0.619
fT4, pmol/L	12.70 (12.10–13.73)	12.10 (11.90–13.70)	0.244	10.10 (8.90–11.90)	10.25 (9.35–11.62)	0.663	12.80 (11.05–14.12)	12.30 (11.90–14.20)	0.874
TSH, mIU/L	1.89 (1.32–2.88)	1.92 (1.22–3.06)	0.773	6.71 (4.52–15.70)	5.84 (4.37–12.84)	0.548	2.46 (1.26–4.14)	3.07 (1.39–4.32)	0.655
TgAb, IU/mL	274.50 (157.62–405.25)	305.00 (139.00–694.00)	0.647	123.00 (30.40–970.50)	265.00 (54.60–730.50)	0.576	131.00 (79.90–422.10)	221.00 (96.10–493.00)	0.334
TPOAb, IU/mL	358.95 (103.55–607.97)	416.00 (231.00–612.00)	0.550	430.00 (27.65–1144.50)	362.15 (62.80–702.75)	0.734	327.50 (131.50–1094.50)	312.00 (136.00–622.00)	0.426
Vitamin D, ng/mL	15.15 (12.30–17.02)	25.30 (23.30–29.90)	<0.001	14.30 (10.95–17.15)	24.85 (22.55–30.52)	<0.001	14.85 (11.83–17.12)	25.00 (21.80–29.30)	<0.001
Disease duration (years)	0.04 (0.00–2.26)	0.02 (0.00–3.61)	0.974	0.00 (0.00–0.05)	0.00 (0.00–0.04)	0.942	6.50 (3.81–10.15)	4.73 (2.15–7.19)	0.081

*p*-Values were calculated using the Wilcoxon rank-sum test. Abbreviations: deficient, patients with HT with Vitamin D deficiency; non-deficient, patients with HT without Vitamin D deficiency; EUTHY, euthyroid patients with HT; LT4, patients with HT receiving levothyroxine therapy; HYPO, hypothyroid patients with HT; BMI, body mass index; BSA, body surface area; T3, triiodothyronine; T4, thyroxine; fT4, free thyroxine; TSH, thyroid-stimulating hormone; TgAb, thyroglobulin antibodies; TPOAb, thyroid peroxidase antibodies. Data are shown as median (IQR).

**Table 3:** Meta-analysis of associations between Vitamin D levels and inflammatory proteomic biomarkers in HT patients stratified by Vitamin D status.

Biomarker	Estimate	SE	p*	I^2^	Tau^2^	*p* ^FDR^	Role of Biomarker
Deficient							
GDNF	−0.027	0.009	0.007	0.208	9.28 × 10^−7^	0.622	Neurotrophic factor; inflammation-associated
IL-17C	−0.051	0.022	0.022	0	0	0.977	Pro-inflammatory cytokine
CCL-11	−0.026	0.012	0.040	0	0	0.977	Eosinophil chemoattractant
CCL-20	−0.057	0.030	0.049	0	0	0.977	Key chemokine guiding Th17 and immune cells
Non-deficient							
CD6	0.023	0.011	0.038	0	0	0.987	T-cell costimulatory receptor

Abbreviations: SE, standard error; p*, nominal *p*-value; *p*^FDR^, false discovery rate (FDR)-adjusted *p*-value; I^2^, heterogeneity index; Tau^2^, between-study variance. None of the associations remained statistically significant after FDR correction. Notes: Estimates (β coefficients) indicate the association between Vitamin D and protein levels; negative values denote inverse associations. Meta-analysis was conducted across HT subgroups stratified by Vitamin D status.

## Data Availability

The data supporting the findings of this study are available from the corresponding author upon reasonable request. The datasets generated and analyzed during the current study are not publicly available but may be accessed through the Croatian Biobank of Patients with Hashimoto’s Thyroiditis (CRO-HT), subject to approval.

## Supplementary Materials

The following supporting information can be downloaded at: https://www.mdpi.com/article/10.3390/diseases14090349/s1, Table S1: Associations between inflammatory proteins and vitamin D levels across study groups; Table S2: Meta-analysis of associations between serum 25(OH)D levels and inflammatory proteomic biomarkers in vitamin D-deficient patients with Hashimoto’s thyroiditis; Table S3: Meta-analysis of associations between serum 25(OH)D levels and inflammatory proteomic biomarkers with relative effect-size estimates in Vitamin D deficient patients with HT; Table S4: Pooled sensitivity analysis of associations between vitamin D status and protein levels.

## Abbreviations

The following abbreviations are used in this manuscript:

25(OH)D	25-hydroxyvitamin D
ALCAM	Activated Leukocyte Cell Adhesion Molecule
BMI	Body Mass Index
BSA	Body Surface Area
CCL11	C-C Motif Chemokine Ligand 11
CCL20	C-C Motif Chemokine Ligand 20
CD6	Cluster of Differentiation 6
CROHT (CRO-HT)	Croatian Biobank of Patients with Hashimoto’s Thyroiditis
ETA	European Thyroid Association
EUTHY	Euthyroid Hashimoto’s thyroiditis subgroup
FDR	False Discovery Rate
fT4	Free Thyroxine
GDNF	Glial Cell Line-Derived Neurotrophic Factor
HT	Hashimoto’s Thyroiditis
HYPO	Hypothyroid Hashimoto’s thyroiditis subgroup
IL-17C	Interleukin-17C
LT4	Levothyroxine Treatment Group
NPX	Normalized Protein eXpression
PEA	Proximity Extension Assay
qPCR	Quantitative Polymerase Chain Reaction
REML	Restricted Maximum Likelihood
SE	Standard Error
T3	Triiodothyronine
T4	Thyroxine
Tg	Thyroglobulin
TgAb	Thyroglobulin Antibodies
Th17	T Helper 17 Cells
TPO	Thyroid Peroxidase
TPOAb	Thyroid Peroxidase Antibodies
TSH	Thyroid-Stimulating Hormone
UVB	Ultraviolet B
VDR	Vitamin D Receptor
