# Supplementary material for: Association of Vitamin D Levels and Systemic Inflammation in Patients with Hashimoto’s Thyroiditis
Source: Diseases. 2026 Sep 20;14(9):349. doi: 10.3390/diseases14090349 (PMC13605515; doi:10.3390/diseases14090349)
Supplement: Supplementary file 1 [file diseases-14-00349-s001.zip › diseases-4472918-supplementary.pdf]

Supplementary Table S1. Associations between inflammatory proteins and Vitamin D levels across study groups

**EUTHY_Vitamin D deficient (*n*=22)**			
Protein	*β*	SE	*P*
CCL-23	0,094	0,030	0,033
FGF-5	0,064	0,020	0,037
**HYPO_Vitamin D deficient (*n*=67)**			
GDNF	-0,030	0,011	0,049
**EUTHY_Vitamin D non-deficient (*n*=25)**			
INF-γ	0,174	0,044	0,018
**LT4_Vitamin D non-deficient (*n*=45)**			
IL-2RB	0,073	0,023	0,032

Abbreviations: EUTHY-euthyroid patients with HT, LT4-patients with HT that were on levothyroxine therapy, HYPO-hypothyroid patients with HT, *β*- beta coefficient, SE- standard error, *P*- P value

Supplementary Table S2. Meta-analysis of associations between serum 25(OH)D levels and inflammatory proteomic biomarkers in Vitamin D-deficient patients with Hashimoto's thyroiditis.

**Assay**	**Estimate**	**SE**	***p*** *	**I2**	**Tau2**	***p*** **FDR**
GDNF	-0,02685	0,009914	0,006759	0,208115	9,28E-07	0,621795068
IL8	-0,03305	0,023528	0,160168	24,74255	0,000442	0,977484895
VEGFA	-0,00071	0,014547	0,960941	0,381496	3,02E-06	0,977484895
CD8A	-0,00912	0,024607	0,711035	59,43204	0,001049	0,977484895
MCP-3	0,007523	0,015525	0,627972	0	0	0,977484895
CDCP1	0,003213	0,012451	0,796374	0	0	0,977484895
CD244	-0,01042	0,015726	0,507568	0	0	0,977484895
IL7	-0,02208	0,020168	0,273533	0	0	0,977484895
OPG	-0,01215	0,009583	0,204643	0	0	0,977484895
LAP TGF-be	-0,0076	0,026068	0,770516	36,91777	0,000759	0,977484895
uPA	0,013515	0,008744	0,122191	0	0	0,977484895
IL6	-0,03461	0,01993	0,082469	0	0	0,977484895
IL-17C	-0,0509	0,022222	0,021999	0	0	0,977484895
MCP-1	-0,01128	0,012904	0,382142	0	0	0,977484895
IL-17A	-0,01678	0,018537	0,365274	0,02797	3,66E-07	0,977484895
CXCL11	0,001907	0,032059	0,952577	0	0	0,977484895
AXIN1	-0,00207	0,052821	0,96869	0	0	0,977484895
TRAIL	-0,00262	0,010662	0,805692	0	0	0,977484895
IL-20RA	-0,00063	0,014413	0,965235	0,51102	4,98E-06	0,977484895
CXCL9	0,016608	0,021698	0,444017	0	0	0,977484895
CST5	0,013312	0,013799	0,334698	0	0	0,977484895
IL-2RB	-0,00343	0,017273	0,842795	0	0	0,977484895
IL-1 alpha	0,003271	0,012387	0,791736	0	0	0,977484895
OSM	-0,00149	0,026547	0,955343	12,23616	0,000301	0,977484895
IL2	-0,01007	0,009225	0,27513	0	0	0,977484895
CXCL1	-0,0279	0,033669	0,407248	17,04031	0,000637	0,977484895
TSLP	-0,01152	0,039321	0,769519	75,31261	0,003471	0,977484895
CCL4	-0,01109	0,018458	0,548019	0	0	0,977484895
CD6	-0,00394	0,017595	0,822771	0	0	0,977484895
SCF	0,002287	0,013083	0,861217	28,23991	0,000155	0,977484895
IL18	0,013082	0,019524	0,502829	0	0	0,977484895
SLAMF1	-0,01145	0,010964	0,296451	0,663684	2,67E-06	0,977484895
TGF-alpha	0,018276	0,015355	0,233973	28,234	0,000206	0,977484895
MCP-4	-0,02217	0,023284	0,340996	0	0	0,977484895
CCL11	-0,02609	0,012688	0,039739	0	0	0,977484895
TNFSF14	-0,01007	0,021803	0,644133	0	0	0,977484895
FGF-23	-0,03103	0,034682	0,370912	0	0	0,977484895
IL-10RA	0,029286	0,03197	0,35964	54,13977	0,001676	0,977484895
FGF-5	0,011757	0,025487	0,6446	91,91189	0,001757	0,977484895
MMP-1	-0,03835	0,046393	0,408496	42,43883	0,00275	0,977484895
LIF-R	0,008488	0,014036	0,545331	51,7148	0,000307	0,977484895
FGF-21	-0,06832	0,049611	0,168462	20,66704	0,001621	0,977484895
CCL19	-0,01722	0,024297	0,478541	0	0	0,977484895
IL-15RA	-0,01094	0,007238	0,130526	7,682591	1,6E-05	0,977484895
IL-10RB	0,000493	0,0077	0,94897	0	0	0,977484895
IL-22 RA1	-0,00558	0,022418	0,803505	39,95754	0,000608	0,977484895
IL-18R1	-0,01371	0,012227	0,262144	0	0	0,977484895

PD-L1	0,010339	0,018506	0,576377	26,77439	0,000288	0,977484895
Beta-NGF	0,000624	0,003382	0,853539	0	0	0,977484895
CXCL5	-0,01346	0,057013	0,813382	38,50388	0,003818	0,977484895
TRANCE	0,012738	0,027284	0,640603	45,62071	0,001015	0,977484895
HGF	-0,01434	0,01098	0,191466	0	0	0,977484895
IL-12B	-0,01407	0,016956	0,406564	0	0	0,977484895
IL-24	-0,00118	0,014636	0,935695	0	0	0,977484895
IL13	-0,04932	0,042688	0,247975	10,68942	0,000715	0,977484895
ARTN	-0,00506	0,009406	0,590707	0,050795	1,67E-07	0,977484895
MMP-10	-0,00942	0,020742	0,649636	0	0	0,977484895
IL10	-0,01475	0,024059	0,539941	27,65314	0,000499	0,977484895
TNF	0,020422	0,032227	0,526287	70,51016	0,002018	0,977484895
CCL23	0,035828	0,025326	0,157169	72,20308	0,001376	0,977484895
CD5	-0,00388	0,011358	0,732667	0,287207	1,61E-06	0,977484895
CCL3	-0,0164	0,016993	0,334396	0	0	0,977484895
Flt3L	-0,00331	0,012054	0,783333	0	0	0,977484895
CXCL6	-0,02337	0,04215	0,57934	39,78623	0,002166	0,977484895
CXCL10	-0,004	0,032852	0,903207	18,11295	0,000667	0,977484895
4E-BP1	-0,00826	0,030283	0,785105	0	0	0,977484895
IL-20	-0,00039	0,013681	0,977485	60,53728	0,00034	0,977484895
SIRT2	-0,02049	0,05004	0,682264	0	0	0,977484895
CCL28	-0,01307	0,019572	0,504127	0	0	0,977484895
DNER	-0,00407	0,007252	0,574319	0	0	0,977484895
EN-RAGE	0,027079	0,029869	0,364616	32,69696	0,000928	0,977484895
CD40	-0,00964	0,023442	0,681056	6,625916	0,000125	0,977484895
IL33	-0,00632	0,007515	0,400731	0	0	0,977484895
IFN-gamma	0,029736	0,030885	0,335638	0	0	0,977484895
FGF-19	-0,00979	0,025577	0,70203	0	0	0,977484895
IL4	0,001739	0,021495	0,935521	0	0	0,977484895
LIF	-0,01951	0,012642	0,122774	0	0	0,977484895
NRTN	0,011159	0,010089	0,268679	0	0	0,977484895
MCP-2	-0,00647	0,027162	0,811766	0	0	0,977484895
CASP-8	0,024227	0,031383	0,440117	0	0	0,977484895
CCL25	0,010476	0,014886	0,481575	0	0	0,977484895
CX3CL1	0,005557	0,016087	0,729782	33,55257	0,000277	0,977484895
TNFRSF9	-0,00035	0,011661	0,976105	0	0	0,977484895
NT-3	-0,01051	0,015179	0,488618	0	0	0,977484895
TWEAK	-0,01455	0,010415	0,162483	0	0	0,977484895
CCL20	-0,05745	0,029171	0,048922	0	0	0,977484895
ST1A1	0,035218	0,031359	0,261409	0	0	0,977484895
STAMBP	-0,00391	0,051074	0,938929	11,71316	0,001013	0,977484895
IL5	-0,01678	0,023723	0,479256	0	0	0,977484895
ADA	-0,00752	0,01747	0,66707	11,46372	0,000112	0,977484895
TNFB	0,00314	0,012023	0,793968	0	0	0,977484895
CSF-1	-0,00944	0,007483	0,207014	0	0	0,977484895

Abbreviations: SE, standard error; *p* *, nominal P-value; *p* FDR, false discovery rate (FDR)-adjusted P-value; I^2, heterogeneity index; τ^2, between-study variance. Estimates (β coefficients) indicate the association between Vitamin D and protein levels.

Supplementary Table S2. Meta-analysis of associations between serum 25(OH)D levels and inflammatory proteomic biomarkers in Vitamin D-non deficient patients with Hashimoto's thyroiditis.

Assay	Estimate	SE	*P* *	I2	Tau2	*p* FDR
IFN-gamma	0,064412	0,04975	0,195421	84,05653	0,006175	0,987246829
CCL4	0,012958	0,01418	0,360813	38,65694	0,000232	0,987246829
SLAMF1	-0,0059	0,010835	0,585913	51,30076	0,000179	0,987246829
CCL11	-0,00085	0,012387	0,945155	69,30419	0,000317	0,987246829
PD-L1	0,005909	0,014268	0,678784	58,09196	0,000352	0,987246829
MMP-10	-0,02726	0,014705	0,063797	0	0	0,987246829
CCL3	0,004865	0,01697	0,774352	57,9849	0,000499	0,987246829
TWEAK	-0,00822	0,006335	0,194665	9,135541	1,25E-05	0,987246829
CDCP1	0,011931	0,007917	0,131831	0,522093	1,05E-06	0,987246829
IL6	0,008167	0,01357	0,547295	22,88093	0,000127	0,987246829
IL18	0,018126	0,010188	0,075223	0,434628	1,37E-06	0,987246829
IL-18R1	0,007506	0,007065	0,288011	5,920413	9,21E-06	0,987246829
IL10	-0,01262	0,029045	0,663887	73,47292	0,001716	0,987246829
CXCL10	0,01866	0,013978	0,181895	0	0	0,987246829
ADA	0,008903	0,009712	0,359324	6,965088	2,12E-05	0,987246829
CSF-1	0,006136	0,004631	0,185222	0	0	0,987246829
MCP-4	-0,01169	0,014331	0,414565	0	0	0,987246829
IL8	0,009152	0,012411	0,460879	0	0	0,987246829
VEGFA	0,007086	0,008977	0,429945	0	0	0,987246829
CD8A	0,008186	0,012227	0,503203	26,09179	0,000118	0,987246829
MCP-3	-0,00652	0,010294	0,52637	0	0	0,987246829
GDNF	0,006688	0,006564	0,308258	7,497249	9,82E-06	0,987246829
CD244	-0,00735	0,009071	0,417883	0	0	0,987246829
IL7	-0,00125	0,01224	0,918799	0	0	0,987246829
OPG	0,010999	0,006422	0,086782	0	0	0,987246829
LAP TGF-be	-0,00024	0,012531	0,985011	0	0	0,987246829
uPA	0,001751	0,005328	0,742346	0	0	0,987246829
IL-17C	0,013034	0,010572	0,217609	0	0	0,987246829
MCP-1	0,002261	0,006711	0,736205	0	0	0,987246829
IL-17A	-0,00317	0,01262	0,801936	0	0	0,987246829
CXCL11	0,011991	0,018279	0,511825	0	0	0,987246829
AXIN1	-0,00796	0,034754	0,818795	0	0	0,987246829
TRAIL	0,00186	0,005166	0,718759	0	0	0,987246829
IL-20RA	-0,00718	0,012196	0,556059	0	0	0,987246829
CXCL9	0,012253	0,010024	0,221592	0	0	0,987246829
CST5	0,004956	0,009546	0,603653	1,113739	3,48E-06	0,987246829
IL-2RB	0,024398	0,020359	0,230775	80,79812	0,00098	0,987246829
OSM	0,018719	0,019396	0,334485	8,286767	9,57E-05	0,987246829
IL2	-0,0019	0,005786	0,742484	0	0	0,987246829
CXCL1	-0,00733	0,022525	0,744778	0	0	0,987246829
TSLP	-0,00394	0,016532	0,811592	0	0	0,987246829
CD6	0,022985	0,011097	0,038338	0	0	0,987246829
SCF	0,001939	0,005438	0,721444	0	0	0,987246829
TGF-alpha	0,002546	0,009027	0,777877	48,41752	0,000119	0,987246829
TNFSF14	0,010604	0,016911	0,530619	0	0	0,987246829
FGF-23	0,003047	0,017153	0,859004	0	0	0,987246829
IL-10RA	0,002262	0,011811	0,848128	0	0	0,987246829

FGF-5	0,005157	0,003828	0,17794	0	0	0,987246829
LIF-R	0,0025	0,005113	0,624906	0	0	0,987246829
FGF-21	0,006729	0,022846	0,768351	0	0	0,987246829
CCL19	0,00052	0,013042	0,968224	0	0	0,987246829
IL-15RA	-0,00063	0,004485	0,888274	0	0	0,987246829
IL-10RB	0,000184	0,005515	0,973443	0	0	0,987246829
IL-22 RA1	-0,0012	0,00805	0,88173	0	0	0,987246829
Beta-NGF	-0,00051	0,002992	0,863686	45,11647	1,22E-05	0,987246829
CXCL5	0,0118	0,027717	0,670296	0	0	0,987246829
TRANCE	-0,0103	0,011207	0,358255	0	0	0,987246829
HGF	0,009101	0,00745	0,221864	0	0	0,987246829
IL-12B	-0,00111	0,01102	0,919651	0	0	0,987246829
IL-24	-0,0007	0,009189	0,939524	0,169413	5,97E-07	0,987246829
ARTN	0,00456	0,006538	0,485569	0	0	0,987246829
TNF	-0,00065	0,011884	0,956215	50,41524	0,000213	0,987246829
CCL23	0,012216	0,009295	0,188762	0	0	0,987246829
CD5	0,008647	0,006842	0,206314	0	0	0,987246829
Flt3L	0,005469	0,007824	0,484584	0	0	0,987246829
CXCL6	-0,01464	0,020186	0,468182	0	0	0,987246829
4E-BP1	0,00989	0,021993	0,652947	0	0	0,987246829
SIRT2	0,002423	0,035654	0,945816	0	0	0,987246829
CCL28	0,000374	0,009979	0,970131	16,5881	5,37E-05	0,987246829
DNER	-0,00056	0,005622	0,919998	0	0	0,987246829
EN-RAGE	-0,00196	0,016812	0,907085	41,34931	0,000351	0,987246829
CD40	0,003018	0,01868	0,87165	36,67097	0,000386	0,987246829
IL33	-0,00702	0,005146	0,172554	0	0	0,987246829
FGF-19	-0,00269	0,020528	0,89583	0	0	0,987246829
IL4	0,027275	0,019443	0,160657	53,67497	0,000607	0,987246829
LIF	0,005843	0,007248	0,420161	0	0	0,987246829
NRTN	-0,00316	0,013901	0,820419	0	0	0,987246829
MCP-2	0,003711	0,013972	0,790534	0	0	0,987246829
CASP-8	0,008719	0,020033	0,663398	0	0	0,987246829
CCL25	-0,00029	0,010105	0,976976	0	0	0,987246829
TNFRSF9	-0,00015	0,007019	0,982862	0	0	0,987246829
NT-3	-0,00256	0,012261	0,834576	0	0	0,987246829
CCL20	-0,00413	0,016572	0,803129	0	0	0,987246829
ST1A1	0,005883	0,026781	0,826118	29,16326	0,00065	0,987246829
STAMBP	0,000524	0,03276	0,987247	0	0	0,987246829
IL5	0,011741	0,014801	0,427609	0	0	0,987246829
TNFB	0,006077	0,009558	0,524916	0	0	0,987246829
IL-1 alpha	-0,00237	0,004733	0,616969	0	0	0,987246829
MMP-1	-0,0087	0,02134	0,683665	0	0	0,987246829
IL13	0,004766	0,024971	0,848646	72,3134	0,00123	0,987246829
IL-20	0,000142	0,004104	0,97231	46,61168	2,34E-05	0,987246829
CX3CL1	-0,0021	0,008669	0,809	0	0	0,987246829

Abbreviations: SE, standard error; *p* *, nominal P-value; *p* FDR, false discovery rate (FDR)-adjusted P-value; I^2, heterogeneity index; τ^2, between-study variance. Estimates (β coefficients) indicate the association between Vitamin D and protein levels.

Supplementary Table S3. Meta-analysis of associations between serum 25(OH)D levels and inflammatory proteomic biomarkers with relative effect-size estimates in Vitamin D deficient patients with HT

Assay	Estimate	SE	CI_95_low	CI_95_high	*p* *	I2	Tau2	Percent change per 10ng	Percent change 10ng CI low	Percent change 10ng CI high	*p* FDR
GDNF	-0,02685	0,00991	-0,0462837	-0,00742114	0,006759	0,208115	9,28E-07	-16,98316952	-27,44419488	-5,013883696	0,6217951
IL-17C	-0,0509	0,02222	-0,0944497	-0,00734234	0,021999	0	0	-29,7271313	-48,03893998	-4,961983581	0,9774849
CCL11	-0,02609	0,01269	-0,0509587	-0,00122416	0,039739	0	0	-16,54410657	-29,7576363	-0,844934857	0,9774849
CCL20	-0,05745	0,02917	-0,1146191	-0,00027155	0,048922	0	0	-32,84592045	-54,81833223	-0,188049191	0,9774849
IL6	-0,03461	0,01993	-0,0736698	0,00445283	0,082469	0	0	-21,32837198	-39,98885547	3,134594464	0,9774849
uPA	0,01351	0,00874	-0,0036228	0,03065259	0,122191	0	0	9,820633736	-2,479840083	23,67259861	0,9774849
LIF	-0,01951	0,01264	-0,0442886	0,00526842	0,122774	0	0	-12,64880992	-26,43383131	3,719284885	0,9774849
IL-15RA	-0,01094	0,00724	-0,0251314	0,00324228	0,130526	7,682591	1,6E-05	-7,305572018	-15,9868972	2,272820455	0,9774849
CCL23	0,03583	0,02533	-0,0138106	0,08546685	0,157169	72,20308	0,001376	28,18976384	-9,128886479	80,83431485	0,9774849
IL8	-0,03305	0,02353	-0,0791608	0,01306907	0,160168	24,74255	0,000442	-20,47163519	-42,23001054	9,481771916	0,9774849
TWEAK	-0,01455	0,01041	-0,0349599	0,00586558	0,162483	0	0	-9,591606791	-21,51975666	4,14949311	0,9774849
FGF-21	-0,06832	0,04961	-0,165558	0,02891337	0,168462	20,66704	0,001621	-37,72275241	-68,25897896	22,19063658	0,9774849
HGF	-0,01434	0,01098	-0,035863	0,00717773	0,191466	0	0	-9,463376252	-22,00954196	5,101065513	0,9774849
OPG	-0,01215	0,00958	-0,0309361	0,0066266	0,204643	0	0	-8,079897806	-19,30011034	4,70033135	0,9774849
CSF-1	-0,00944	0,00748	-0,0241099	0,00522451	0,207014	0	0	-6,335569883	-15,3899301	3,68772275	0,9774849
TGF-alpha	0,01828	0,01536	-0,0118203	0,04837192	0,233973	28,234	0,000206	13,50518343	-7,866536192	39,83438951	0,9774849
IL13	-0,04932	0,04269	-0,1329829	0,03435009	0,247975	10,68942	0,000715	-28,95348183	-60,21846615	26,88318562	0,9774849
ST1A1	0,03522	0,03136	-0,0262443	0,09668115	0,261409	0	0	27,64916882	-16,6325099	95,45161168	0,9774849
IL-18R1	-0,01371	0,01223	-0,0376741	0,01025361	0,262144	0	0	-9,06561975	-22,98243302	7,365914498	0,9774849
NRTN	0,01116	0,01009	-0,0086144	0,03093306	0,268679	0	0	8,042090867	-5,796257123	23,91326546	0,9774849
IL7	-0,02208	0,02017	-0,0616108	0,01744506	0,273533	0	0	-14,19274538	-34,75711783	12,85345927	0,9774849
IL2	-0,01007	0,00922	-0,0281473	0,00801297	0,27513	0	0	-6,74013568	-17,72495769	5,711307452	0,9774849
SLAMF1	-0,01145	0,01096	-0,0329352	0,01004148	0,296451	0,663684	2,67E-06	-7,627737566	-20,41058696	7,20816426	0,9774849
CCL3	-0,0164	0,01699	-0,049709	0,01690241	0,334396	0	0	-10,74734315	-29,14655279	12,42976974	0,9774849
CST5	0,01331	0,0138	-0,0137339	0,04035797	0,334698	0	0	9,666290984	-9,080570456	32,27860578	0,9774849
IFN-gamma	0,02974	0,03088	-0,0307965	0,09026942	0,335638	0	0	22,88974917	-19,22196371	86,95540452	0,9774849
MCP-4	-0,02217	0,02328	-0,0678081	0,02346524	0,340996	0	0	-14,24540617	-37,50038102	17,66232313	0,9774849
IL-10RA	0,02929	0,03197	-0,0333741	0,09194678	0,35964	54,13977	0,001676	22,50694856	-20,65235009	89,14173844	0,9774849
EN-RAGE	0,02708	0,02987	-0,0314629	0,08562189	0,364616	32,69696	0,000928	20,64723249	-19,59424054	81,02875721	0,9774849

IL-17A	-0,01678	0,01854	-0,0531153	0,01954946	0,365274	0,02797	3,66E-07	-10,98187997	-30,79984236	14,51167114	0,9774849
FGF-23	-0,03103	0,03468	-0,0990067	0,03694281	0,370912	0	0	-19,3536829	-49,6545606	29,18406392	0,9774849
MCP-1	-0,01128	0,0129	-0,0365694	0,01401406	0,382142	0	0	-7,519372214	-22,390475	10,20124806	0,9774849
IL33	-0,00632	0,00752	-0,0210444	0,00841428	0,400731	0	0	-4,28285539	-13,57289319	6,005767296	0,9774849
IL-12B	-0,01407	0,01696	-0,0473054	0,01916023	0,406564	0	0	-9,293728416	-27,95620464	14,20314078	0,9774849
CXCL1	-0,0279	0,03367	-0,0938924	0,0380867	0,407248	17,04031	0,000637	-17,58540995	-47,83780864	30,21241011	0,9774849
MMP-1	-0,03835	0,04639	-0,129274	0,05258265	0,408496	42,43883	0,00275	-23,34014517	-59,1824963	43,97581455	0,9774849
CASP-8	0,02423	0,03138	-0,0372816	0,08573615	0,440117	0	0	18,28544678	-22,77265559	81,17218746	0,9774849
CXCL9	0,01661	0,0217	-0,0259187	0,05913481	0,444017	0	0	12,20059471	-16,44416297	50,66539816	0,9774849
CCL19	-0,01722	0,0243	-0,0648406	0,03040378	0,478541	0	0	-11,25020041	-36,2015294	23,45949445	0,9774849
IL5	-0,01678	0,02372	-0,0632797	0,02971195	0,479256	0	0	-10,98247766	-35,50751237	22,86887317	0,9774849
CCL25	0,01048	0,01489	-0,0186992	0,0396514	0,481575	0	0	7,531611946	-12,15648505	31,63234161	0,9774849
NT-3	-0,01051	0,01518	-0,0402613	0,0192384	0,488618	0	0	-7,026876909	-24,35129747	14,26503468	0,9774849
IL18	0,01308	0,01952	-0,0251849	0,05134928	0,502829	0	0	9,491745354	-16,0180391	42,75020698	0,9774849
CCL28	-0,01307	0,01957	-0,0514342	0,0252859	0,504127	0	0	-8,66382093	-29,98879603	19,15660829	0,9774849
CD244	-0,01042	0,01573	-0,0412436	0,02040222	0,507568	0	0	-6,968363894	-24,8646171	15,19053983	0,9774849
TNF	0,02042	0,03223	-0,0427424	0,08358628	0,526287	70,51016	0,002018	15,2062763	-25,64117846	78,49242127	0,9774849
IL10	-0,01475	0,02406	-0,0619008	0,03240906	0,539941	27,65314	0,000499	-9,716065534	-34,88816007	25,18750556	0,9774849
LIF-R	0,00849	0,01404	-0,0190208	0,03599742	0,545331	51,7148	0,000307	6,060165927	-12,35209018	28,34029721	0,9774849
CCL4	-0,01109	0,01846	-0,0472655	0,02508882	0,548019	0	0	-7,39791131	-27,93627585	18,99394503	0,9774849
DNER	-0,00407	0,00725	-0,0182864	0,01013981	0,574319	0	0	-2,783916438	-11,9047771	7,281264437	0,9774849
PD-L1	0,01034	0,01851	-0,0259318	0,04660956	0,576377	26,77439	0,000288	7,429405066	-16,451707	38,1365993	0,9774849
CXCL6	-0,02337	0,04215	-0,1059785	0,05924681	0,57934	39,78623	0,002166	-14,95246021	-52,02965544	50,78240717	0,9774849
ARTN	-0,00506	0,00941	-0,0234931	0,01337622	0,590707	0,050795	1,67E-07	-3,445500543	-15,02745934	9,715106697	0,9774849
MCP-3	0,00752	0,01552	-0,0229048	0,03795075	0,627972	0	0	5,352891866	-14,68020967	30,08976911	0,9774849
TRANCE	0,01274	0,02728	-0,0407375	0,06621255	0,640603	45,62071	0,001015	9,230479726	-24,60057259	58,24122425	0,9774849
TNFSF14	-0,01007	0,0218	-0,0528051	0,03266209	0,644133	0	0	-6,74294467	-30,65091778	25,40725976	0,9774849
FGF-5	0,01176	0,02549	-0,038197	0,06171013	0,6446	91,91189	0,001757	8,490260705	-23,26110941	53,37903085	0,9774849
MMP-10	-0,00942	0,02074	-0,050077	0,03123174	0,649636	0	0	-6,322541599	-29,3270384	24,17006467	0,9774849
ADA	-0,00752	0,01747	-0,0417563	0,02672581	0,66707	11,46372	0,000112	-5,07580701	-25,13115429	20,35182765	0,9774849
CD40	-0,00964	0,02344	-0,0555814	0,03631075	0,681056	6,625916	0,000125	-6,460554041	-31,97268382	28,61933179	0,9774849
SIRT2	-0,02049	0,05004	-0,1185622	0,07759177	0,682264	0	0	-13,23724162	-56,03650677	71,22789131	0,9774849
FGF-19	-0,00979	0,02558	-0,0599154	0,04034483	0,70203	0	0	-6,557746245	-33,98591987	32,26655237	0,9774849

CD8A	-0,00912	0,02461	-0,0573445	0,03911246	0,711035	59,43204	0,001049	-6,123245388	-32,79897378	31,14152494	0,9774849
CX3CL1	0,00556	0,01609	-0,025974	0,03708779	0,729782	33,55257	0,000277	3,926873263	-16,47617701	29,31394421	0,9774849
CD5	-0,00388	0,01136	-0,02614	0,01838096	0,732667	0,287207	1,61E-06	-2,653250656	-16,57221657	13,58793459	0,9774849
TSLP	-0,01152	0,03932	-0,08859	0,06554732	0,769519	75,31261	0,003471	-7,675429076	-45,88501864	57,51324646	0,9774849
LAP TGF-be	-0,0076	0,02607	-0,0586974	0,04348905	0,770516	36,91777	0,000759	-5,134318912	-33,42622699	35,18082335	0,9774849
Flt3L	-0,00331	0,01205	-0,0269404	0,02031113	0,783333	0	0	-2,271338891	-17,03377744	15,11782635	0,9774849
4E-BP1	-0,00826	0,03028	-0,06761	0,0510956	0,785105	0	0	-5,562762023	-37,41453574	42,49941297	0,9774849
IL-1 alpha	0,00327	0,01239	-0,0210068	0,02754837	0,791736	0	0	2,29304166	-13,55033504	21,03998757	0,9774849
TNFB	0,00314	0,01202	-0,0204255	0,02670565	0,793968	0	0	2,200391763	-13,20132243	20,33501395	0,9774849
CDCP1	0,00321	0,01245	-0,0211903	0,02761601	0,796374	0	0	2,251957092	-13,66025072	21,09674647	0,9774849
IL-22 RA1	-0,00558	0,02242	-0,0495173	0,0383613	0,803505	39,95754	0,000608	-3,792599176	-29,05236286	30,46049659	0,9774849
TRAIL	-0,00262	0,01066	-0,0235189	0,01827365	0,805692	0	0	-1,801429994	-15,0426123	13,50347995	0,9774849
MCP-2	-0,00647	0,02716	-0,0597057	0,0467684	0,811766	0	0	-4,384688848	-33,88987774	38,28877355	0,9774849
CXCL5	-0,01346	0,05701	-0,125202	0,09828447	0,813382	38,50388	0,003818	-8,906978092	-58,01400137	97,63585268	0,9774849
CD6	-0,00394	0,01759	-0,0384265	0,03054456	0,822771	0	0	-2,694689737	-23,38306065	23,58002663	0,9774849
IL-2RB	-0,00343	0,01727	-0,0372801	0,03042894	0,842795	0	0	-2,346466944	-22,77183929	23,48102596	0,9774849
Beta-NGF	0,00062	0,00338	-0,0060035	0,00725204	0,853539	0	0	0,433639266	-4,075933825	5,15521598	0,9774849
SCF	0,00229	0,01308	-0,0233551	0,02792956	0,861217	28,23991	0,000155	1,598027599	-14,94610423	21,36021658	0,9774849
CXCL10	-0,004	0,03285	-0,0683845	0,0603941	0,903207	18,11295	0,000667	-2,731264011	-37,74959419	51,98627025	0,9774849
IL4	0,00174	0,02149	-0,04039	0,04386788	0,935521	0	0	1,212637386	-24,41875856	35,5362491	0,9774849
IL-24	-0,00118	0,01464	-0,0298666	0,02750494	0,935695	0	0	-0,815155421	-18,6996302	21,0035504	0,9774849
STAMBP	-0,00391	0,05107	-0,1040171	0,09619079	0,938929	11,71316	0,001013	-2,675930825	-51,37300901	94,78841375	0,9774849
IL-10RB	0,00049	0,0077	-0,014598	0,01558358	0,94897	0	0	0,342146822	-9,623495475	11,40668122	0,9774849
CXCL11	0,00191	0,03206	-0,0609273	0,06474038	0,952577	0	0	1,330300323	-34,44728383	56,6346959	0,9774849
OSM	-0,00149	0,02655	-0,0535168	0,05054369	0,955343	12,23616	0,000301	-1,025124535	-30,99218824	41,95531961	0,9774849
VEGFA	-0,00071	0,01455	-0,0292242	0,02779933	0,960941	0,381496	3,02E-06	-0,492594411	-18,33678496	21,25072178	0,9774849
IL-20RA	-0,00063	0,01441	-0,0288773	0,02762088	0,965235	0,51102	4,98E-06	-0,434488777	-18,14019416	21,10083726	0,9774849
AXIN1	-0,00207	0,05282	-0,1056009	0,10145436	0,96869	0	0	-1,426810414	-51,90393279	102,0263666	0,9774849
TNFRSF9	-0,00035	0,01166	-0,0232043	0,02250577	0,976105	0	0	-0,241808286	-14,85718308	16,88240035	0,9774849
IL-20	-0,00039	0,01368	-0,0272011	0,02642884	0,977485	60,53728	0,00034	-0,267279095	-17,18355109	20,10434823	0,9774849

Abbreviations: SE, standard error; CI, confidence interval; *p* *, nominal *P* -value; I², heterogeneity index; τ², between-study variance; % change per 10 ng/mL, estimated relative percentage difference in protein level associated with a 10 ng/mL higher serum 25(OH)D concentration; *p* FDR, false discovery rate-adjusted *P* -value. Percentage differences and their 95% CIs were derived from the NPX (log2) scale and represent relative rather than absolute differences in protein concentrations.

Supplementary Table S3. Meta-analysis of associations between serum 25(OH)D levels and inflammatory proteomic biomarkers with relative effect-size estimates in Vitamin D non deficient patients with HT

Assay	Estimate	SE	CI_95_low	CI_95_high	*p* *	I2	Tau2	Percent change per 10ng	Percent change 10ng CI low	Percent change 10ng CI high	*p* FDR
CD6	0,022985	0,011097	0,001235	0,044735	0,038338	0	0	17,27106381	0,859489955	36,35308301	0,987246829
MMP-10	-0,02726	0,014705	-0,05608	0,001564	0,063797	0	0	-17,21626756	-32,20753551	1,090090312	0,987246829
IL18	0,018126	0,010188	-0,00184	0,038094	0,075223	0,434628	1,37E-06	13,38700294	-1,269092873	30,21872085	0,987246829
OPG	0,010999	0,006422	-0,00159	0,023586	0,086782	0	0	7,921835905	-1,094990144	17,76069465	0,987246829
CDCP1	0,011931	0,007917	-0,00359	0,027449	0,131831	0,522093	1,05E-06	8,621412905	-2,455611875	20,95633145	0,987246829
IL4	0,027275	0,019443	-0,01083	0,065382	0,160657	53,67497	0,000607	20,81114256	-7,232895413	57,33305715	0,987246829
IL33	-0,00702	0,005146	-0,0171	0,003066	0,172554	0	0	-4,748714633	-11,17998284	2,148228002	0,987246829
FGF-5	0,005157	0,003828	-0,00235	0,012661	0,17794	0	0	3,639543665	-1,613141072	9,172659114	0,987246829
CXCL10	0,01866	0,013978	-0,00874	0,046057	0,181895	0	0	13,80787337	-5,876055885	37,60825858	0,987246829
CSF-1	0,006136	0,004631	-0,00294	0,015213	0,185222	0	0	4,344735166	-2,018192092	11,12087018	0,987246829
CCL23	0,012216	0,009295	-0,006	0,030434	0,188762	0	0	8,836211689	-4,074839444	23,48502631	0,987246829
TWEAK	-0,00822	0,006335	-0,02063	0,0042	0,194665	9,135541	1,25E-05	-5,535523718	-13,32523619	2,954272813	0,987246829
IFN-gamma	0,064412	0,04975	-0,0331	0,16192	0,195421	84,05653	0,006175	56,27814038	-20,49951992	207,2038953	0,987246829
CD5	0,008647	0,006842	-0,00476	0,022057	0,206314	0	0	6,176749178	-3,247858356	16,51940592	0,987246829
IL-17C	0,013034	0,010572	-0,00769	0,033754	0,217609	0	0	9,455245126	-5,188275597	26,36043444	0,987246829
CXCL9	0,012253	0,010024	-0,00739	0,0319	0,221592	0	0	8,863953648	-4,996299163	24,74630251	0,987246829
HGF	0,009101	0,00745	-0,0055	0,023704	0,221864	0	0	6,511838031	-3,741386359	17,85720998	0,987246829
IL-2RB	0,024398	0,020359	-0,01551	0,064301	0,230775	80,79812	0,00098	18,42515696	-10,1901571	56,15791487	0,987246829
IL-18R1	0,007506	0,007065	-0,00634	0,021353	0,288011	5,920413	9,21E-06	5,34057595	-4,299575452	15,95180475	0,987246829
GDNF	0,006688	0,006564	-0,00618	0,019554	0,308258	7,497249	9,82E-06	4,745110482	-4,191572641	14,51537691	0,987246829
OSM	0,018719	0,019396	-0,0193	0,056734	0,334485	8,286767	9,57E-05	13,85445553	-12,51886304	48,17865307	0,987246829
TRANCE	-0,0103	0,011207	-0,03226	0,01167	0,358255	0	0	-6,888003046	-20,03838467	8,425072971	0,987246829
ADA	0,008903	0,009712	-0,01013	0,027939	0,359324	6,965088	2,12E-05	6,365469998	-6,782769445	21,36826143	0,987246829
CCL4	0,012958	0,01418	-0,01483	0,040749	0,360813	38,65694	0,000232	9,397175809	-9,771135071	32,63762194	0,987246829
MCP-4	-0,01169	0,014331	-0,03978	0,016396	0,414565	0	0	-7,785040382	-24,09935018	12,03591534	0,987246829
CD244	-0,00735	0,009071	-0,02513	0,01043	0,417883	0	0	-4,965879404	-15,98417273	7,497412942	0,987246829
LIF	0,005843	0,007248	-0,00836	0,02005	0,420161	0	0	4,133405335	-5,632266242	14,90968019	0,987246829
IL5	0,011741	0,014801	-0,01727	0,04075	0,427609	0	0	8,478767025	-11,28046788	32,63869426	0,987246829
VEGFA	0,007086	0,008977	-0,01051	0,02468	0,429945	0	0	5,033938322	-7,025551909	18,65763579	0,987246829

IL8	0,009152	0,012411	-0,01517	0,033478	0,460879	0	0	6,54925572	-9,983295829	26,11819105	0,987246829
CXCL6	-0,01464	0,020186	-0,05421	0,02492	0,468182	0	0	-9,652221298	-31,32206368	18,85507274	0,987246829
Flt3L	0,005469	0,007824	-0,00987	0,020803	0,484584	0	0	3,86327719	-6,610111012	15,5112236	0,987246829
ARTN	0,00456	0,006538	-0,00826	0,017375	0,485569	0	0	3,211070102	-5,561587963	12,79864582	0,987246829
CD8A	0,008186	0,012227	-0,01578	0,03215	0,503203	26,09179	0,000118	5,837816895	-10,36033263	24,96301931	0,987246829
CXCL11	0,011991	0,018279	-0,02383	0,047816	0,511825	0	0	8,666489606	-15,22835039	39,29664006	0,987246829
TNFB	0,006077	0,009558	-0,01266	0,024809	0,524916	0	0	4,301957479	-8,398720889	18,76360723	0,987246829
MCP-3	-0,00652	0,010294	-0,0267	0,013655	0,52637	0	0	-4,420116201	-16,89472383	9,927005943	0,987246829
TNFSF14	0,010604	0,016911	-0,02254	0,043748	0,530619	0	0	7,626948381	-14,46426919	35,42364002	0,987246829
IL6	0,008167	0,01357	-0,01843	0,034763	0,547295	22,88093	0,000127	5,823874345	-11,99218587	27,24656887	0,987246829
IL-20RA	-0,00718	0,012196	-0,03108	0,016723	0,556059	0	0	-4,85474623	-19,38202581	12,29033483	0,987246829
SLAMF1	-0,0059	0,010835	-0,02714	0,015334	0,585913	51,30076	0,000179	-4,008756238	-17,14769572	11,21379132	0,987246829
CST5	0,004956	0,009546	-0,01375	0,023665	0,603653	1,113739	3,48E-06	3,494660675	-9,092823236	17,82507355	0,987246829
IL-1 alpha	-0,00237	0,004733	-0,01164	0,006909	0,616969	0	0	-1,627335276	-7,753230481	4,905366501	0,987246829
LIF-R	0,0025	0,005113	-0,00752	0,012522	0,624906	0	0	1,747898791	-5,080154922	9,067128164	0,987246829
4E-BP1	0,00989	0,021993	-0,03322	0,052996	0,652947	0	0	7,095534919	-20,56563946	44,38907195	0,987246829
CASP-8	0,008719	0,020033	-0,03054	0,047983	0,663398	0	0	6,229790849	-19,08097895	39,4575505	0,987246829
IL10	-0,01262	0,029045	-0,06955	0,044306	0,663887	73,47292	0,001716	-8,376942108	-38,25026224	35,94850832	0,987246829
CXCL5	0,0118	0,027717	-0,04252	0,066125	0,670296	0	0	8,523227173	-25,52874852	58,14546691	0,987246829
PD-L1	0,005909	0,014268	-0,02206	0,033873	0,678784	58,09196	0,000352	4,180528772	-14,17650518	26,46400148	0,987246829
MMP-1	-0,0087	0,02134	-0,05052	0,03313	0,683665	0	0	-5,848997687	-29,5437175	25,81434787	0,987246829
TRAIL	0,00186	0,005166	-0,00826	0,011985	0,718759	0	0	1,297849928	-5,567688638	8,662535651	0,987246829
SCF	0,001939	0,005438	-0,00872	0,012597	0,721444	0	0	1,352896267	-5,864627996	9,123800788	0,987246829
MCP-1	0,002261	0,006711	-0,01089	0,015414	0,736205	0	0	1,579414747	-7,272088631	11,27585371	0,987246829
uPA	0,001751	0,005328	-0,00869	0,012194	0,742346	0	0	1,221441362	-5,846106777	8,819506455	0,987246829
IL2	-0,0019	0,005786	-0,01324	0,009439	0,742484	0	0	-1,309017507	-8,769209959	6,761215387	0,987246829
CXCL1	-0,00733	0,022525	-0,05148	0,036815	0,744778	0	0	-4,955514742	-30,01106591	29,06974931	0,987246829
FGF-21	0,006729	0,022846	-0,03805	0,051506	0,768351	0	0	4,774539676	-23,18183353	42,90505318	0,987246829
CCL3	0,004865	0,01697	-0,02839	0,038125	0,774352	57,9849	0,000499	3,429659981	-17,86607982	30,24697398	0,987246829
TGF-alpha	0,002546	0,009027	-0,01515	0,020239	0,777877	48,41752	0,000119	1,78070952	-9,966299104	15,06039102	0,987246829
MCP-2	0,003711	0,013972	-0,02367	0,031096	0,790534	0	0	2,605769149	-15,13350006	24,05299937	0,987246829
IL-17A	-0,00317	0,01262	-0,0279	0,021569	0,801936	0	0	-2,170301275	-17,58366225	16,12563982	0,987246829
CCL20	-0,00413	0,016572	-0,03661	0,028349	0,803129	0	0	-2,823054238	-22,41339339	21,71377509	0,987246829

CX3CL1	-0,0021	0,008669	-0,01909	0,014895	0,809	0	0	-1,441875048	-12,39127341	10,87598658	0,987246829
TSLP	-0,00394	0,016532	-0,03634	0,028462	0,811592	0	0	-2,694602365	-22,26873545	21,80865015	0,987246829
AXIN1	-0,00796	0,034754	-0,07608	0,060154	0,818795	0	0	-5,369216876	-40,98209965	51,73337347	0,987246829
NRTN	-0,00316	0,013901	-0,0304	0,02409	0,820419	0	0	-2,163613403	-19,00067993	18,17331966	0,987246829
ST1A1	0,005883	0,026781	-0,04661	0,058373	0,826118	29,16326	0,00065	4,162286554	-27,60627424	49,8718546	0,987246829
NT-3	-0,00256	0,012261	-0,02659	0,02147	0,834576	0	0	-1,759133225	-16,83265123	16,0463577	0,987246829
IL-10RA	0,002262	0,011811	-0,02089	0,025411	0,848128	0	0	1,580158701	-13,47851011	19,25971981	0,987246829
IL13	0,004766	0,024971	-0,04418	0,053707	0,848646	72,3134	0,00123	3,358421692	-26,37653359	45,10269424	0,987246829
FGF-23	0,003047	0,017153	-0,03057	0,036666	0,859004	0	0	2,134550843	-19,09630247	28,93683223	0,987246829
Beta-NGF	-0,00051	0,002992	-0,00638	0,005351	0,863686	45,11647	1,22E-05	-0,355420134	-4,324507728	3,778324636	0,987246829
CD40	0,003018	0,01868	-0,03359	0,03963	0,87165	36,67097	0,000386	2,113960966	-20,77339565	31,61312554	0,987246829
IL-22 RA1	-0,0012	0,00805	-0,01697	0,014579	0,88173	0	0	-0,826683866	-11,10015364	10,63401159	0,987246829
IL-15RA	-0,00063	0,004485	-0,00942	0,00816	0,888274	0	0	-0,435776084	-6,320921391	5,819088224	0,987246829
FGF-19	-0,00269	0,020528	-0,04292	0,037546	0,89583	0	0	-1,84573152	-25,73320098	29,7249989	0,987246829
EN-RAGE	-0,00196	0,016812	-0,03491	0,030989	0,907085	41,34931	0,000351	-1,350917789	-21,49456221	23,96136746	0,987246829
IL7	-0,00125	0,01224	-0,02524	0,022743	0,918799	0	0	-0,861226945	-16,0493191	17,07464689	0,987246829
IL-12B	-0,00111	0,01102	-0,02271	0,020488	0,919651	0	0	-0,767578663	-14,56553806	15,25879863	0,987246829
DNER	-0,00056	0,005622	-0,01158	0,010455	0,919998	0	0	-0,390637177	-7,715523684	7,515646813	0,987246829
IL-24	-0,0007	0,009189	-0,01871	0,017313	0,939524	0,169413	5,97E-07	-0,482069181	-12,1615104	12,75032847	0,987246829
CCL11	-0,00085	0,012387	-0,02513	0,023427	0,945155	69,30419	0,000317	-0,588921959	-15,9866186	17,63081395	0,987246829
SIRT2	0,002423	0,035654	-0,06746	0,072304	0,945816	0	0	1,693765495	-37,34827876	65,06524858	0,987246829
TNF	-0,00065	0,011884	-0,02394	0,02264	0,956215	50,41524	0,000213	-0,451241227	-15,29306952	16,9910811	0,987246829
CCL19	0,00052	0,013042	-0,02504	0,026081	0,968224	0	0	0,360756373	-15,93457464	19,81479159	0,987246829
CCL28	0,000374	0,009979	-0,01919	0,019932	0,970131	16,5881	5,37E-05	0,259336529	-12,45179923	14,8160039	0,987246829
IL-20	0,000142	0,004104	-0,0079	0,008187	0,97231	46,61168	2,34E-05	0,098797253	-5,329745066	5,838620784	0,987246829
IL-10RB	0,000184	0,005515	-0,01063	0,010994	0,973443	0	0	0,127348904	-7,100882414	7,917989525	0,987246829
CCL25	-0,00029	0,010105	-0,0201	0,019513	0,976976	0	0	-0,201932927	-13,00298811	14,48271585	0,987246829
TNFRSF9	-0,00015	0,007019	-0,01391	0,013606	0,982862	0	0	-0,104451248	-9,189911339	9,890000192	0,987246829
LAP TGF-be	-0,00024	0,012531	-0,0248	0,024325	0,985011	0	0	-0,163048362	-15,79138194	18,36575807	0,987246829
STAMBP	0,000524	0,03276	-0,06368	0,064732	0,987247	0	0	0,363623846	-35,68820649	56,62534725	0,987246829

Abbreviations: SE, standard error; CI, confidence interval; *p* *, nominal *P* -value; I², heterogeneity index; τ², between-study variance; % change per 10 ng/mL, estimated relative percentage difference in protein level associated with a 10 ng/mL higher serum 25(OH)D concentration; *p* FDR, false discovery rate-adjusted P-value. Percentage differences and their 95% CIs were derived from the NPX (log2) scale and represent relative rather than absolute differences in protein concentrations.

Supplementary Table S4. Pooled sensitivity analysis of associations between vitamin D status and protein levels

Protein	VitD_status	*n* _total	*n* _EUTHY	*n* _HYPO	*n* _LT4	Beta_VitaminD	SE	CI_low	CI_high	*P* _value	FDR
IL-17C	Deficient	137	22	67	48	-0,05603449	0,022398	-0,10037	-0,0117	0,013662	0,666486
CCL11		137	22	67	48	-0,027573653	0,011119	-0,04958	-0,00557	0,014489	0,666486
IL6		137	22	67	48	-0,039587041	0,018669	-0,07654	-0,00264	0,035962	0,88138
GDNF		137	22	67	48	-0,019627653	0,009818	-0,03906	-0,0002	0,04777	0,88138
CCL20		137	22	67	48	-0,055493646	0,027774	-0,11047	-0,00052	0,047901	0,88138
IFN-gamma	Non deficient	120	25	50	45	0,057325988	0,017746	0,022146	0,092506	0,001643	0,151154
OPG		120	25	50	45	0,011753224	0,005827	0,000203	0,023304	0,046184	0,990341

Abbreviations: VitD, vitamin D; EUTHY, euthyroid patients with HT; HYPO, hypothyroid patients with HT; LT4, pateints that were take levothyroxine; n, number of participants; β, regression coefficient; SE, standard error; CI, confidence interval; FDR, false discovery rate.
